# 10,12 Conjugated Linoleic Acid-Driven Weight Loss Is Protective against Atherosclerosis in Mice and Is Associated with Alternative Macrophage Enrichment in Perivascular Adipose Tissue

**DOI:** 10.3390/nu10101416

**Published:** 2018-10-03

**Authors:** Jenny E. Kanter, Leela Goodspeed, Shari Wang, Farah Kramer, Tomasz Wietecha, Diego Gomes-Kjerulf, Savitha Subramanian, Kevin D. O’Brien, Laura J. den Hartigh

**Affiliations:** 1 Department of Medicine, Division of Metabolism, Endocrinology and Nutrition, University of Washington Medicine Diabetes Institute, University of Washington, Box 358062, 750 Republican Street, Seattle, WA 98109, USA; jenka@u.washington.edu (J.E.K.); leelag@u.washington.edu (L.G.); sawang@u.washington.edu (S.W.); fkramer@u.washington.edu (F.K.); tomaszw@u.washington.edu (T.W.); gkjerulf@u.washington.edu (D.G.-K.); ssubrama@u.washington.edu (S.S.); cardiac@u.washington.edu (K.D.O.); 2 Department of Medicine, Cardiology, University of Washington, Box 356422, 1959 Pacific Ave NE, Seattle, WA 98195, USA

**Keywords:** alternatively activated macrophages, perivascular adipose tissue, type 2 cytokines

## Abstract

The dietary fatty acid 10,12 conjugated linoleic acid (10,12 CLA) promotes weight loss by increasing fat oxidation, but its effects on atherosclerosis are less clear. We recently showed that weight loss induced by 10,12 CLA in an atherosclerosis-susceptible mouse model with characteristics similar to human metabolic syndrome is accompanied by accumulation of alternatively activated macrophages within subcutaneous adipose tissue. The objective of this study was to evaluate whether 10,12 CLA-mediated weight loss was associated with an atheroprotective phenotype. Male low-density lipoprotein receptor deficient (Ldlr^−/−^) mice were made obese with 12 weeks of a high-fat, high-sucrose diet feeding (HFHS: 36% fat, 36% sucrose, 0.15% added cholesterol), then either continued on the HFHS diet with or without caloric restriction (CR), or switched to a diet with 1% of the lard replaced by either 9,11 CLA or 10,12 CLA for 8 weeks. Atherosclerosis and lipid levels were quantified at sacrifice. Weight loss in mice following 10,12 CLA supplementation or CR as a weight-matched control group had improved cholesterol and triglyceride levels, yet only the 10,12 CLA-treated mice had improved en face and aortic sinus atherosclerosis. 10,12 CLA-supplemented mice had increased lesion macrophage content, with enrichment of surrounding perivascular adipose tissue (PVAT) alternative macrophages, which may contribute to the anti-atherosclerotic effect of 10,12 CLA.

## 1. Introduction

Conjugated linoleic acids (CLAs), of which the major isomers are cis-9, trans-11 conjugated linoleic acid (9,11 CLA) and trans-10, cis-12 CLA (10,12 CLA), are microbial metabolites found naturally in ruminant animal food products and are major components of widely used CLA weight loss supplements [1,2,3]. Commercial CLA supplements differ significantly in the ratio of 9,11 CLA to 10,12 CLA (approximately 1:1) when compared to levels found in food (approximately 5:1). As such, CLA supplement users ingest significantly more 10,12 CLA than would be obtained from the diet. Supplemental CLA promotes significant weight loss in animals and modest weight loss in humans, an effect now attributed to the 10,12 CLA isomer [4]. We have previously shown that 10,12 CLA reduces lipid accumulation by increasing fatty acid oxidation in a cell line derived from (mouse) 3T3 cells (3T3-L1) adipocytes [5]. Moreover, obese mice supplemented with 10,12 CLA lose body weight and fat mass due to enhanced fatty acid oxidation with increased energy expenditure and white adipose tissue browning [4]. These changes in energy expenditure are not the result of weight loss per se, as a control group undergoing caloric restriction to mirror weight loss by the 10,12 CLA-supplemented group did not exhibit these changes [4]. While the weight loss properties of 10,12 CLA are well established, if and how 10,12 CLA impacts atherosclerosis is less well-defined.

Studies of CLA supplementation in small animal models susceptible to the development of atherosclerosis have yielded mixed results. Initial studies in rabbits suggested that mixed CLA supplementation, an approximately equal mixture of the two most common isomers, 9,11 CLA and 10,12 CLA, was protective against atherosclerosis [6,7]. Subsequent studies have shown a similar effect in atherosclerosis-prone mice that lack the apolipoprotein-E gene (ApoE^−/−^) when fed a diet containing mixed CLA [8,9], or a CLA diet containing 80% 9,11 CLA and 20% 10,12 CLA [10,11,12]. While these studies have shown atheroprotective effects of mixed CLA supplementation, there is also abundant evidence that CLA-containing diets do not provide protection from atherosclerosis, and may even contribute to its development [13,14,15,16]. Additional studies could clarify the impact of supplemental CLA on atherosclerosis.

Most previous CLA supplementation studies with the end point of atherosclerosis assessment utilized ApoE^−/−^ mice. However, while these mice are an efficient model with which to study atherosclerosis, they poorly replicate human atherosclerotic lipoprotein profiles and co-morbidities such as obesity and insulin resistance. While ApoE^−/−^ mice develop hypercholesterolemia due to elevated low-density lipoprotein (LDL) and very-low-density lipoprotein (VLDL), they also exhibit decreased high-density lipoprotein (HDL) [17]. Conversely, low-density lipoprotein receptor deficient (Ldlr^−/−^) mice accumulate LDL and HDL cholesterol with more modest VLDL elevation, which more closely resembles dyslipidemic humans [18]. Humans inclined to take CLA supplements would likely be obese with characteristics of metabolic syndrome, including insulin resistance, hepatic steatosis, and systemic inflammation. As such, a better mouse model with which to study the effects of CLA supplementation on atherosclerosis would include these phenotypes, as well as the propensity to develop atherosclerosis. We therefore utilized Ldlr^−/−^ mice consuming a diet high in saturated fat and refined carbohydrates, which has previously been shown to promote a phenotype closely resembling human metabolic syndrome [19], to study the effects of 10,12 CLA on atherosclerosis. In addition, and in contrast to previous studies, mice in this study were supplemented with individual isomers of CLA rather than mixed CLA in order to clearly identify effects due to 9,11 or 10,12 CLA alone.

## 2. Materials and Methods

### 2.1. Mouse Study Design

**Figure 1:**
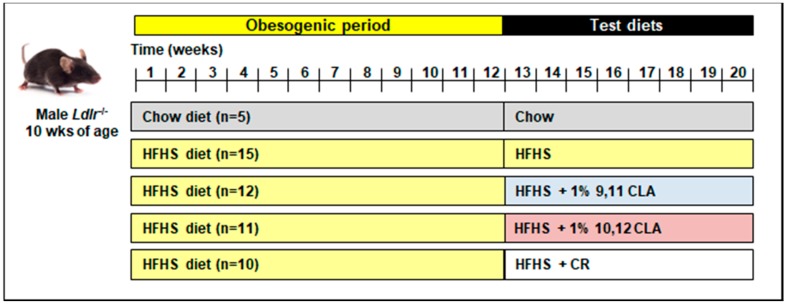
**Study schematic.** 10-week male low-density lipoprotein receptor deficiency (*Ldlr*^−/−^) mice were fed either a chow or obesogenic high-fat, high-sucrose (HFHS) diet for 12 weeks, then either maintained on those diets or switched to a HFHS diet containing 1% 9,11 conjugated linoleic acid (9,11 CLA) (*w*/*w*), 1% 10,12 conjugated linoleic acid (10,12 CLA) (*w*/*w*), or calorically restricted (CR) on the HFHS diet to consume 85% of baseline food intake for an additional 8 weeks. wks: weeks.

Ten-week-old adult male Ldlr^−/−^ mice were randomized into treatment groups, and fed either normal chow or a high-fat, high-sucrose ((HFHS): 36% fat from lard, 36.2% sucrose diet with 0.15% added cholesterol) for 12 weeks (chow: *n* = 5; HFHS: *n* = 10–15). Mice were then switched to one of five test diets for an additional 8 weeks: (1) chow → chow diet; (2) HFHS → HFHS diet; (3) HFHS → HFHS + 1% 9,11 CLA; (4) HFHS → HFHS + 1% 10,12 CLA; (5) HFHS → HFHS + caloric restriction (CR). The study design is shown in Figure 1. CLA diets replaced 1% lard with 1% of either CLA isomer (>90%purity, Nu-Check Prep, Waterville, MN, USA). All test diets were prepared by BioServ (Flemington, NJ, USA) and have been previously described [4]. CR was begun at 85% total food intake per mouse and adjusted daily to mirror weight loss by 10,12 CLA, ending at an average of 74.4% CR after 8 weeks. HFHS, 9,11 CLA, and 10,12 CLA diets were fed ad libitum. Mice were individually housed for the duration of test diet feeding. Body weights were recorded weekly, and body composition, glucose and insulin tolerance, energy intake, and energy expenditure for these exact mice have been previously reported [4], with relevant phenotypes shown in Table S1. As such, this study adheres strongly to the “Reduction” component of the “Replacement, Reduction and Refinement (3Rs)” of animal research, as the same animals were utilized for multiple studies. At sacrifice, blood was collected and phosphate buffered saline (PBS)-perfused harvested tissues were snap-frozen in liquid nitrogen and stored at −70 °C or were fixed with 10% neutral-buffered formalin and embedded in paraffin wax. All experimental procedures were undertaken with approval from the Institution Animal Care and Use Committee of the University of Washington (#3104-01 03/15/13–02/28/19) and followed the guidelines of the National Institutes of Health guide for the care and use of laboratory animals (NIH Publications No. 8023, revised 1978).

### 2.2. Plasma Analyses

Triglycerides and cholesterol were measured from fasting plasma, and pooled plasma fast-phase liquid chromatography (FPLC) fractions using colorimetric assays as previously described [20]. Lipids were extracted from plasma using the Bligh and Dyer method [21], the fatty acid components were derivatized into methyl esters, and fatty acid compositions were quantified using gas chromatography as previously described [4]. Serum amyloid A (SAA) was quantified from plasma using enzyme-linked immunosorbent assay (ELISA) [22].

### 2.3. Atherosclerosis

Aortas were perfused with saline, the perivascular adipose tissue (PVAT) surrounding the thoracic aorta was completely removed and collected, and the thoracic aorta was excised down to the level of the diaphragm. PVAT samples were flash frozen and stored at −80 °C, and aortas were fixed in 4% formalin. Atherosclerosis from the aortic arch was quantified using the en face method using Sudan IV staining as described previously [23,24], and from the aortic sinus using Movat’s pentachrome staining as described previously [25]. Quantification for total lesion size and necrotic core areas was performed blinded on digital images of stained tissue sections using Image Pro Plus analysis software Version 6 (Media Cybernetics, Inc., Rockville, MD, USA). Necrotic cores were identified and outlined for quantification using Image J software, as in shown in Figure S1.

### 2.4. Immunohistochemistry

Formalin-fixed, paraffin-embedded hearts were sectioned through the aortic sinus and stained with Movat’s pentachrome for lesion quantification, Picro-Sirius Red for collagen quantification, and a rat monoclonal Galectin-3 (MAC2) antibody (1:3000 dilution, Cedarlane Laboratories, Burlington, NC, USA) for relative quantification of atherosclerotic plaque macrophages. To further characterize plaque macrophage phenotypes, sequential sections were also stained with rabbit polyclonal antibodies against CD206 (Mannose receptor) to identify resident macrophages (1:100 dilution, AbCam, Cambridge, MA, USA). Area quantification for MAC2 and Cluster of Differentiation 206 (CD206) staining was performed on digital images of immunostained tissue sections using image analysis software Image Pro Plus software Version 6 (Media Cybernetics, Inc., Rockville, MD, USA).

### 2.5. Quantitative Real-Time PCR

Aortas were perfused through the left ventricle with RNA-later (Thermo Fisher Scientific, Waltham, MA, USA), excised from the heart to the diaphragm, snap frozen in liquid nitrogen and stored at −80 °C until processed. PVAT was completely removed from the aortas prior to freezing. Total RNA was extracted and purified using a commercially available kit (Qiagen RNeasy Mini Kit (Qiagen, Hilden, Nordrhein-Westfalen, Germany)). After spectroscopic quantification, 2 µg of RNA was reverse-transcribed, and the cDNA thus obtained was analyzed by real-time quantitative polymerase chain reaction (RT-PCR) by standard protocols using an ABI 7900HT instrument (Thermo Fisher Scientific, Waltham, MA, USA). Primer and probe set for individual genes (TaqMan system) were purchased from Thermo Fisher Scientific. Glyceraldehyde-3-Phosphate Dehydrogenase (GAPDH) was used as a housekeeping gene, levels of which did not change with the various treatments. Relative amounts of the target gene were calculated using the ΔΔCt formula and expressed as a fold change from HFHS-fed control mice. Accession numbers for Taqman primers used are shown in Table S2.

### 2.6. Bone Marrow-Derived Macrophage Culture

Bone marrow was isolated from donor C57Bl/6 male mice (*n* = 3) and differentiated into bone marrow-derived macrophages (BMDMs) in Roswell Park Memorial Institute (RPMI)-1640 medium (GE Life Sciences, Pittsburgh, PA, USA) that contained 30% L-cell conditioned medium over the course of 7 days. Non-polarized BMDMs were treated with media alone (control), lipopolysaccharides (LPS) (10 ng/mL for 4 h), interleukin-4 (IL-4) (10 ng/mL for 24 h), 9,11 CLA or 10,12 CLA (100 µM for 24 h, conjugated to bovine serum albumin (BSA, Sigma-Aldrich, St. Louis, MO, USA) as described previously [5]). In a separate experiment, BMDMs were treated for 24 h with 10% *v*/*v* serum that had been isolated from mice fed the HFHS diet with or without 1% 9,11 CLA or 1% 10,12 CLA. Total RNA was extracted from >1 × 10^6^ macrophages and reverse transcribed for RT-PCR analysis as described above. To determine if CLA isomers influenced cholesterol loading, BMDMs were loaded with acetylated low-density lipoprotein (Ac-LDL, 50 µg/mL) in the presence or absence of 9,11 CLA or 10,12 CLA (100 µM) for 24 h. Intracellular cholesterol was quantified using an Amplex Red assay (Thermo Fisher Scientific), presented normalized to total protein content (bicinchoninic acid (BCA) assay, Thermo Fisher Scientific, Waltham, MA, USA).

### 2.7. Statistics

Data were analyzed using GraphPad Prism 6 software (GraphPad Software Inc., California, CA, USA) and are represented as means ± standard errors. One-way analysis of variance (ANOVA) was used to compare differences between mice receiving the different diets as indicated, and Bonferroni post-hoc testing was used to detect differences among mean values of the groups. A *p* value < 0.05 was considered statistically significant.

## 3. Results

### 3.1. Weight Loss by 10,12 CLA and CR Improves Plasma Triglycerides, Cholesterol, Fatty Acids, and Lipoprotein Profiles

**Figure 2:**
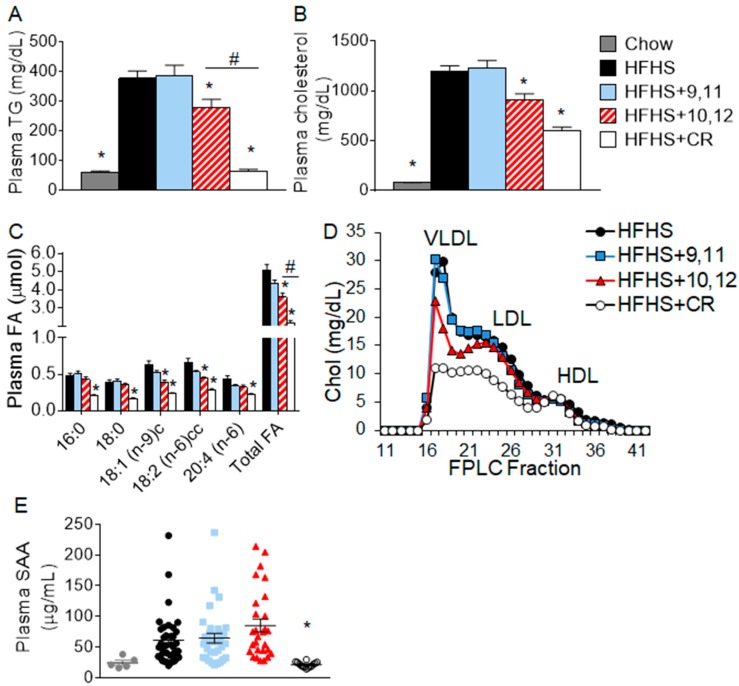
Fasting plasma lipid levels were decreased by 10,12 conjugated linoleic acid (CLA) and caloric restriction (CR). (**A**) Triglycerides (TG), (**B**) cholesterol, (**C**) fatty acids (FA), (**D**) fast phase liquid chromatography (FPLC)-fractionated cholesterol, and (**E**) serum amyloid A (SAA) were quantified from plasma following a 4-year. Fast in mice that had been fed chow or high-fat, high-sucrose (HFHS) diet for 12 weeks followed by 8 weeks of the indicated diets. Plasma samples were pooled prior to FPLC fractionation. Data are presented as mean ± standard error of mean (SEM), *n* = 5–15 mice/group. * *p* < 0.05 from HFHS control; ^#^ *p* < 0.05 from 10,12 CLA. LDL, low-density lipoprotein; VLDL, very-low-density lipoprotein; HDL, high-density lipoprotein.

We previously reported that mice supplemented with 10,12 CLA lost significant body weight and body fat, while mice calorically restricted to lose equivalent body weight lost mass equally from lean and fat compartments [4]. Control obese mice consuming the HFHS diet with or without 9,11 CLA remained obese [4]. Metabolic parameters such as glucose tolerance and energy expenditure have been reported previously [4]. Consistent with weight loss, 10,12 CLA supplementation improved plasma triglyceride (TG), cholesterol, and fatty acid (FA) levels when compared with obese HFHS-fed control mice with and without 9,11 CLA, as seen in Figure 2A–C. Circulating lipoprotein profiles measured using FPLC were similarly improved by 10,12 CLA, as seen in Figure 2D, with reduced cholesterol content of VLDL and LDL-containing fractions. While mice that had undergone CR lost equivalent body weight as 10,12 CLA-supplemented mice, they exhibited further improvements in plasma triglycerides (TG), cholesterol, fatty acids (FA), and lipoprotein profiles, as shown in Figure 2A–D. Moreover, plasma serum amyloid A (SAA), an acute phase reactant that becomes chronically elevated during obesity [26], was also improved by CR, as shown in Figure 2E, but not 10,12 CLA. Supplementation with 9,11 CLA had no effect on plasma lipids or SAA. Collectively, weight loss by both 10,12 CLA and CR resulted in improvements in plasma lipid levels, with significantly lower lipid levels and SAA seen following CR-mediated weight loss.

### 3.2. Weight Loss Following 10,12 CLA Supplementation Improves Atherosclerosis

**Figure 3:**
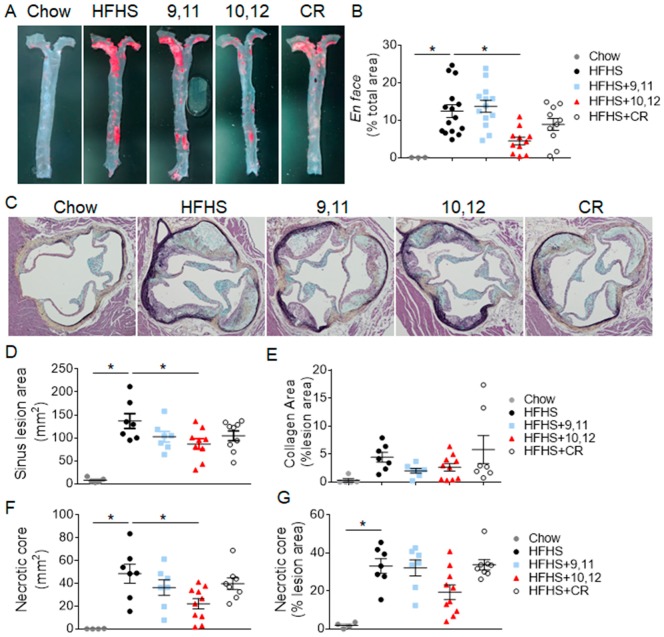
Atherosclerosis lesion size was decreased by 10,12 CLA, but not CR. Mice were fed either chow or HFHS diet for 12 weeks, then continued on the indicated diets for an additional 8 weeks. (**A**,**B**) Aortas were prepared en face (**A**), stained with Sudan IV, and lesions imaged and quantified using Image Pro Plus software (**B**). (**C**–**G**) Sections through the aortic sinus of the heart were stained with Movat’s pentachrome, and lesion area (**C**), collagen (**D**), and necrotic core area (**F**–**G**) was quantified using Image Pro Plus software. Data are presented as mean ± SEM, *n* = 5–10 mice/group. * *p* < 0.05 from HFHS control.

To determine if weight loss-mediated reductions in plasma lipids improved atherosclerosis, the extent of thoracic aortic atherosclerosis was evaluated using the en face technique. Obese mice consuming the HFHS diet with or without 9,11 CLA, shown in Figure 3A,B, displayed extensive atherosclerosis compared to chow-fed control mice, evident by increased Sudan IV staining. In contrast, mice supplemented with 10,12 CLA had significantly reduced levels of atherosclerosis, with no significant reductions following CR, despite having greater reductions in circulating lipid levels and systemic inflammation. To determine if the anti-atherogenic effect of 10,12 CLA was not an effect exclusive to the thoracic aorta, atherosclerotic lesions were quantified from the aortic sinus. Similarly, obese mice fed the HFHS diet with or without 9,11 CLA had extensive lesion development in the aortic sinus, as seen in Figure 3C,D. Also consistent with the en face analysis, 10,12 CLA supplementation significantly reduced aortic sinus lesion size, shown in Figure 3C,D, with no significant reductions following CR. Collagen levels were equivalent among treatment groups, shown in Figure 3E. Further, necrotic core areas represented a smaller proportion of atherosclerotic lesions in 10,12 CLA-treated mice as shown in Figure 3F,G. Taken together, 10,12 CLA supplementation significantly reduced atherosclerotic lesion area from both the aorta and the aortic sinus with reduced necrotic core size, with no significant differences evoked by CR-mediated weight loss or by 9,11 CLA. Thus, improved aortic atherosclerosis appears to be an effect specific to 10,12 CLA supplementation, and not weight loss or reduced circulating lipid levels per se.

### 3.3. Aortic Sinus Lesions Contain More Macrophages Following 10,12 CLA Supplementation

**Figure 4:**
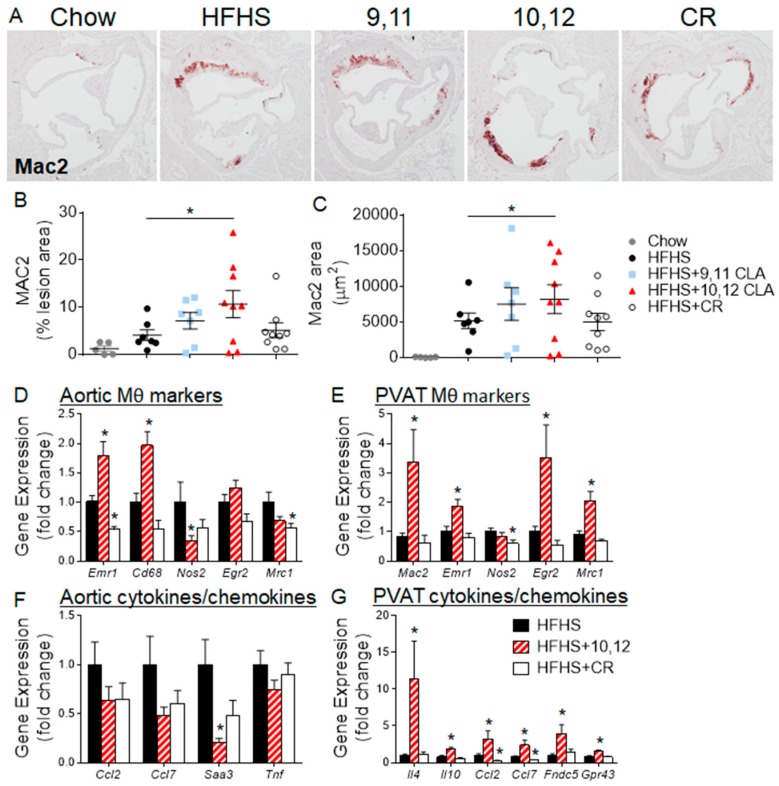
10,12 CLA increases macrophage content of atherosclerotic lesions. Mice were fed either chow or HFHS diet for 12 weeks, then continued on the indicated diets for an additional 8 weeks. (**A**) Sections through the aortic sinus of the heart were stained with a Mac2 antibody, and (**B**,**C**) quantified using Image Pro Plus software. *n* = 5–10 mice/group. Different letters indicate a significant difference (*p* < 0.05). (**D**–**G**) Gene expression was quantified from the aortic arch (**D**,**F**) or the surrounding perivascular adipose tissue (PVAT, **E**,**G**) from a different cohort of different mice. *n* = 8 mice/group. Data are presented as mean ± SEM. * *p* < 0.05 from HFHS control.

To further characterize the aortic sinus lesions in response to different diets, sections from the aortic sinus were stained for Mac2, a general macrophage-specific marker. As expected, obese mice fed the HFHS diet with or without 9,11 CLA or CR, shown in Figure 4A–C, exhibited higher Mac2-positive areas than lean chow-fed mice. However, mice supplemented with 10,12 CLA had significantly elevated Mac2 staining when expressed as total stained area or the percentage of total lesion area, as seen in Figure 4B,C. Gene expression from the aortic arches of a separate cohort of mice confirmed elevation of macrophage-specific genes Emr1 and Cd68 from mice given 10,12 CLA, as seen in Figure 4D. The lack of a corresponding increase in monocyte recruitment chemokines Ccl2 and Saa3 implies that the enrichment in lesion macrophages may not be due to increased monocyte recruitment, as shown in Figure 4F. These results suggest that while 10,12 CLA supplementation results in fewer atherosclerotic lesions, those lesions contain more macrophages.

Macrophages are generally classified based on their function, stratified as “classically”, “alternatively”, or “metabolically” activated [27]. Classically-activated macrophages will be defined herein as those that express Nos2 and secrete pro-inflammatory cytokines such as tumor necrosis factor (TNF), while alternatively-activated, or resident, macrophages express Egr2 and secrete type-2 cytokines such as IL-4 [27]. A previous study in the exact same mice presented herein showed that adipose tissue from 10,12 CLA-treated mice exhibited a significant elevation in gene expression for markers indicative of alternative activation, or resident M2 macrophages, including Arg1 and Egr2 [4]. To determine if the elevation of macrophages in sinus lesions was due to enrichment in M2 macrophages, sinus sections were stained for the mannose receptor, CD206. As shown in Figure S2A, there were no differences in CD206 staining between groups. Gene expression from aortic arches confirmed the lack of enrichment in resident macrophage markers *Egr2* and *Mrc1* (encodes CD206), with a significant reduction in the M1 marker *Nos2*, as seen in Figure 4D, promoting a reduction in the M1/M2 ratio shown in Figure S2B. This suggests that M2 macrophages may predominate in 10,12 CLA-supplemented mouse aortas. In contrast, perivascular adipose tissue (PVAT) surrounding the aortic arches was enriched in the general macrophage markers *Mac2* and *Emr1* as well as resident macrophage markers *Egr2* and *Mrc1* following 10,12 CLA supplementation as seen in Figure 4E. Moreover, PVAT from 10,12 CLA-supplemented mice was enriched with type-2 cytokine expression, including *Il4* and *Il10*, further supporting an M2 phenotype. Expression of chemokines *Ccl2* and *Ccl7* was increased in PVAT in response to 10,12 CLA, shown in Figure 4G, suggesting the recruitment of new macrophages. Finally, *Fndc5*, the gene encoding the browning marker irisin, and *Gpr43*, a major short-chain fatty acid (SCFA) receptor, were also enriched in PVAT from 10,12 CLA-treated mice, as seen in Figure 4G, supporting the known effects of 10,12 CLA to promote the browning of adipose tissue and increases in fecal and systemic SCFA [4,28]. In summary, while 10,12 CLA increased the macrophage content of aortic and sinus lesions and the surrounding PVAT, macrophage polarization towards a resident M2 phenotype was only observed in PVAT.

### 3.4. 10,12 CLA Induces a Resident Alternatively-Activated Macrophage Phenotype

**Figure 5:**
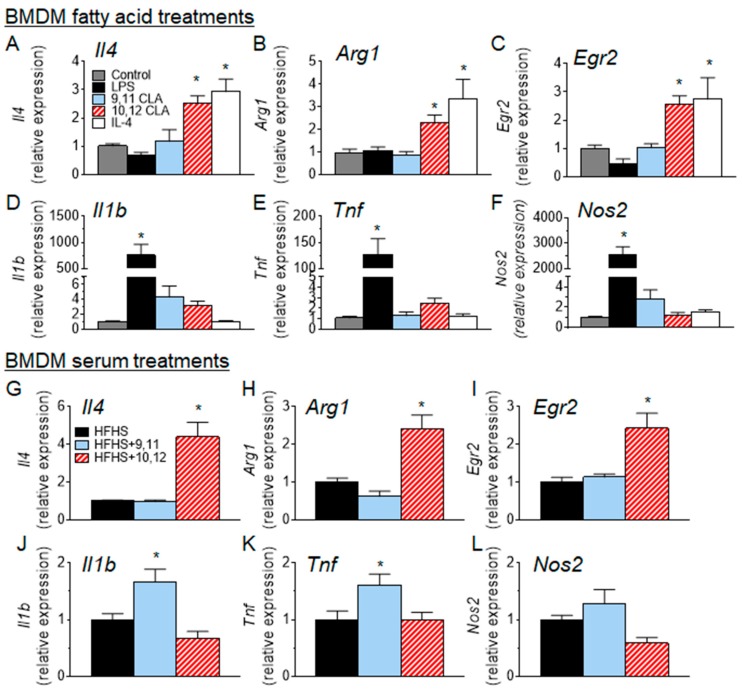
10,12 CLA promotes the alternative polarization of macrophages in vitro. (**A**–**F**) bone marrow-derived macrophages (BMDMs) were treated with media alone (control), lipopolysaccharides (LPS) (10 ng/mL for 4 h), interleukin-4 (IL-4) (10 ng/mL for 24 h), or 10,12 CLA (100 µM for 24 h). (**G**–**L**) BMDMs were treated for 24 h with 10% serum isolated from mice fed a HFHS diet with or without 1% 9,11 or 10,12 CLA. Total RNA was extracted from >1 × 10^6^ macrophages and reverse transcribed for real-time quantitative polymerase chain reaction (RT-PCR) analysis. *n* = 3; data are presented as mean ± SEM. * *p* < 0.05 from media or HFHS control.

To further examine what effect 10,12 CLA has on macrophage polarization in vitro, bone marrow-derived macrophages (BMDMs) were cultured with or without agents known to polarize macrophages towards classically- or alternatively-activated profiles. As shown in Figure 5A–F, BMDMs that have been classically-activated by treatment with lipopolysaccharide (LPS) express high levels of type-1 cytokines *Il1β* and *Tnf* and the classic marker of classically activated macrophages *Nos2*, while BMDMs that have been alternatively-activated by IL-4 treatment express *Il4*, *Arg1*, and *Egr2*, markers that have been previously associated with resident macrophage populations [29]. BMDMs treated with 10,12 CLA express a similar complement of markers as those treated with IL-4, as seen in Figure 5A–F, suggesting that 10,12 CLA promotes an alternative macrophage phenotype. Further, BMDMs treated with 10% serum collected from mice that had been fed a HFHS diet that contained 10,12 CLA, as in Figure 1, exhibited a similar gene expression profile as shown in Figure 5G–L, suggesting that a component of the serum in 10,12 CLA-supplemented mice promotes the alternative activation of macrophages. Co-treatment with 9,11 CLA or 10,12 CLA had no impact on acetylated LDL-mediated cholesterol loading of BMDMs as seen in Figure S3. Collectively, these results suggest that 10,12 CLA: (1) reduces plasma lipid levels and atherosclerotic lesion area, (2) increases atherosclerotic lesion macrophage content, and (3) polarizes the surrounding adipose tissue macrophages towards an alternative phenotype.

## 4. Discussion

While it has been suggested that mixed CLA supplementation may have anti-atherogenic properties in certain mouse models [8,9,10,11,12,30], we now show for the first time that obese HFHS diet-fed Ldlr^−/−^ mice, which represent a more humanized model of metabolic syndrome, are protected from atherosclerosis following CLA supplementation, and that the 10,12 isomer of CLA is responsible for this protection. Further, the anti-atherosclerotic effects of 10,12 CLA may be independent from its weight loss and lipid-lowering effects, as a weight-matched control group undergoing CR-mediated weight loss does not exhibit equivalent atheroprotection. Moreover, a potential mechanism by which 10,12 CLA is atheroprotective may involve its effects on local macrophage populations.

It is well known that dramatic lipid-lowering therapies, such as statins or PCSK9 inhibitors, improve atherosclerosis in humans and mice [31,32], and that atherosclerosis has a strong inflammatory component [33]. It is therefore striking that 10,12 CLA supplementation resulted in reduced atherosclerotic lesion size while CR did not, despite profoundly improved systemic triglyceride, cholesterol, and SAA levels by CR. It is possible that CR-mediated improvements in lipids and SAA were insufficient to modulate atherosclerosis in the time frame of this study. It is also possible that the improvements in systemic lipids and inflammation by CR were trumped by a lack of improvement at the local tissue level.

The reduction of atherosclerosis by 10,12 CLA supplementation was also striking, given the increased macrophage content of those lesions. It has been previously reported that atherosclerosis regression in response to thiazolidinedione treatment occurs despite increased lesion macrophage content [34], which were identified as largely resident M2 macrophages. Moreover, it is becoming increasingly appreciated that M2 macrophages support lesion regression by resolving inflammation and promoting tissue remodeling [34,35,36]. Mice deficient in transcription factors that are required for M2 polarization have accelerated atherosclerosis [37], while administering type-2 cytokines such as IL-4 to mice protects against atherosclerosis [38]. Thus, it is tempting to speculate that the reduction in atherosclerosis in response to 10,12 CLA is due to enhanced M2 macrophage content. Indeed, we previously showed that these exact same mice supplemented with 10,12 CLA had increased markers of alternatively activated macrophages within adipose tissue as seen in Table S1 [4]. The polarization towards an M2 phenotype could explain the increased macrophage content, as IL-4 (which is secreted from M2 macrophages) is a strong stimulus for macrophage proliferation [39]. While we did not observe appreciable changes in alternative macrophage markers directly quantified from atherosclerotic lesions in these mice, it is possible that a more sensitive method such as analysis of laser capture microdissected macrophages would reveal a more robust effect. Nevertheless, the observed decrease in the M1/M2 ratio within the aortas of 10,12 CLA-treated mice suggest that M2 macrophages play a more dominant role, supporting previous observations that the M1/M2 ratio is positively correlated with the severity of coronary artery disease [40]. Notably, and in contrast to the current study, a growing body of work suggests that IL-4 may exert pro-atherogenic effects by accelerating endothelial cell apoptosis and/or enhancing vascular inflammation and oxidative stress [41,42,43,44,45,46,47]. While we did not observe enhanced inflammation (i.e., increased *Tnf*, *Saa3*, or *Ccl2*) in aortic or PVAT tissue from mice supplemented with 10,12 CLA, nor in macrophages treated with IL-4, whether the observed increase in IL-4 is causally associated with improved atherosclerosis in these mice remains to be explored.

The lesion microenvironment, which includes adjacent PVAT, is thought to be important for atherosclerotic progression and/or regression [48]. Mice deficient in PVAT exhibit endothelial dysfunction, and lose cold-mediated protection from atherosclerosis [49]. Our observation that resident macrophages and anti-inflammatory signals are enriched in the PVAT immediately adjacent to the aorta suggests that a signal from the PVAT may confer atheroprotection. It is possible that the “alternative” M2 milieu in the lesion microenvironment (in this case PVAT) promotes regression of atherosclerosis and/or plaque stabilization, as is evidenced by the reduced necrotic core size. Previous observations in humans support this notion, as epicardial adipose tissue M1:M2 macrophage ratios positively correlate with the severity of coronary artery disease [40]. PVAT is a plastic adipose tissue depot, and can possess a phenotype resembling brown adipose tissue [50]. As we have previously shown that 10,12 CLA supplementation promotes the browning and M2 macrophage enrichment of white adipose tissue [4], and we now show that PVAT in these mice expresses elevated levels of irisin and the M2 markers *Egr2* and *Mrc1*, it is plausible that M2 macrophage-containing PVAT secretes factors that promote atherosclerosis regression. Future PVAT transplantation studies could address whether this is the case.

In addition to changes in adipose tissue, 10,12 CLA consumption promotes significant alterations to the gut microbiota, characterized by increased abundance of species that produce SCFAs such as acetate and butyrate [28]. Metabolic diseases such as atherosclerosis are associated with decreased SCFA-producing bacteria such as the butyrate-producing genera *Butyrivibrio* and *Roseburia* [51]. In our previous study using the exact same mice presented herein, *Butyrivibrio* and *Roseburia* species were significantly enriched in the gut microbiota of mice given 10,12 CLA [28]. Atherosclerosis-prone ApoE^−/−^ mice fed a butyrate-supplemented chow diet are protected from atherosclerosis [52,53], and mounting evidence shows that butyrate blunts macrophage inflammation [54,55,56], suggesting that butyrate has anti-atherogenic properties. Further, evidence suggests that butyrate promotes macrophage M2 polarization [57]. Thus, it is tempting to speculate that at least part of the atheroprotection incurred by 10,12 CLA may be related to its effects on the gut microbiota and gut-derived SCFA.

In conclusion, mice supplemented with 10,12 CLA experience reduced atherosclerosis and an anti-inflammatory, M2-like state in the aortic lesion microenvironment. Mice that are calorically restricted with markedly lowered cholesterol, triglyceride, and systemic inflammation do not experience such atheroprotection, suggesting that alterations to the aortic microenvironment may be more effective at impacting atherosclerosis. Future studies will evaluate a causal role for PVAT components and the gut microbiota in promoting atherosclerosis regression.

## Supplementary Materials

The following are available online at http://www.mdpi.com/2072-6643/10/10/1416/s1, Figure S1: Representative image of necrotic cores, Figure S2: 10,12 CLA decreases the ratio of M1/M2 macrophages, Figure S3: CLA isomers do not alter the uptake of cholesterol by bone marrow-derived macrophages, Table S1: Phenotype summary of study mice, Table S2: Taqman primers.

## Abbreviations

CLA	conjugated linoleic acid
HFHS	high fat high sucrose
PVAT	perivascular adipose tissue
LDLR	low-density lipoprotein receptor
CR	caloric restriction
SAA	serum amyloid A
BMDM	bone marrow-derived macrophages
FPLC	fast phase liquid chromatography
TG	triglycerides
FA	fatty acids
SCFA	short-chain fatty acids
