# Supplementary material for: 10,12 Conjugated Linoleic Acid-Driven Weight Loss Is Protective against Atherosclerosis in Mice and Is Associated with Alternative Macrophage Enrichment in Perivascular Adipose Tissue
Source: Nutrients. 2018 Oct 3;10(10):1416. doi: 10.3390/nu10101416 (PMC6213611; doi:10.3390/nu10101416)
Supplement: Supplementary file 1 [file nutrients-10-01416-s001.pdf]

Supplementary Material

Table S1. Phenotype summary of study mice[1].

**Table 1**	**Body weight (g)**	**Adiposity (% BW)**	**Fasting glucose (mg/dL)**	**Liver TG (mg/mg protein)**	**IWAT *Egr2* expression**
**Chow**	28±0.7*#	11±2.2*#	156±16*#	--	--
**HFHS**	48±1.0#	43±3.8#	270±12#	1.60±0.09	1.0±0.1
**9,11 CLA**	47±1.3#	40±1.8#	240±08#	1.31±0.16	0.99±0.03
**10,12 CLA**	35±0.7*	23±1.3*	207±09*	**2.15±0.14***	**12.7±0.4***
**CR**	34±0.6*	37±2.3#	144±15*#	0.96±0.10*#	0.8±0.1

*P<0.05 from HFHS control mice.
#P<0.05 from 10,12 CLA-supplemented mice.
-- not measured

Figure S1.

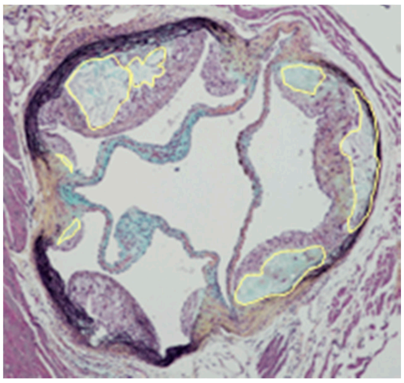

Figure S1. Representative image of necrotic core areas identified for quantification using Image J software.

Table S2. Taqman primers

Gene name	Thermo Fisher Scientific accession number
Emr1	Mm00802530_m1
Cd68	Mm00839636_g1
Mac2	Mm00802901_m1
Nos2	Mm00440502_m1
Egr2	Mm00456650_m1
Mrc1	Mm01329362_m1
Arg1	Mm01190441_g1
Ccl2	Mm00441242_m1
Ccl7	Mm00443113_m1
Saa3	Mm00441203_m1
Tnf	Mm00443258_m1
Il1b	Mm00434228_m1
Il4	Mm00445259_m1
Il10	Mm01288368_m1
Fndc5	Mm01181543_m1
Gpr43	Mm01176527_m1
Gapdh	Mm00662311_g1

Figure S2.

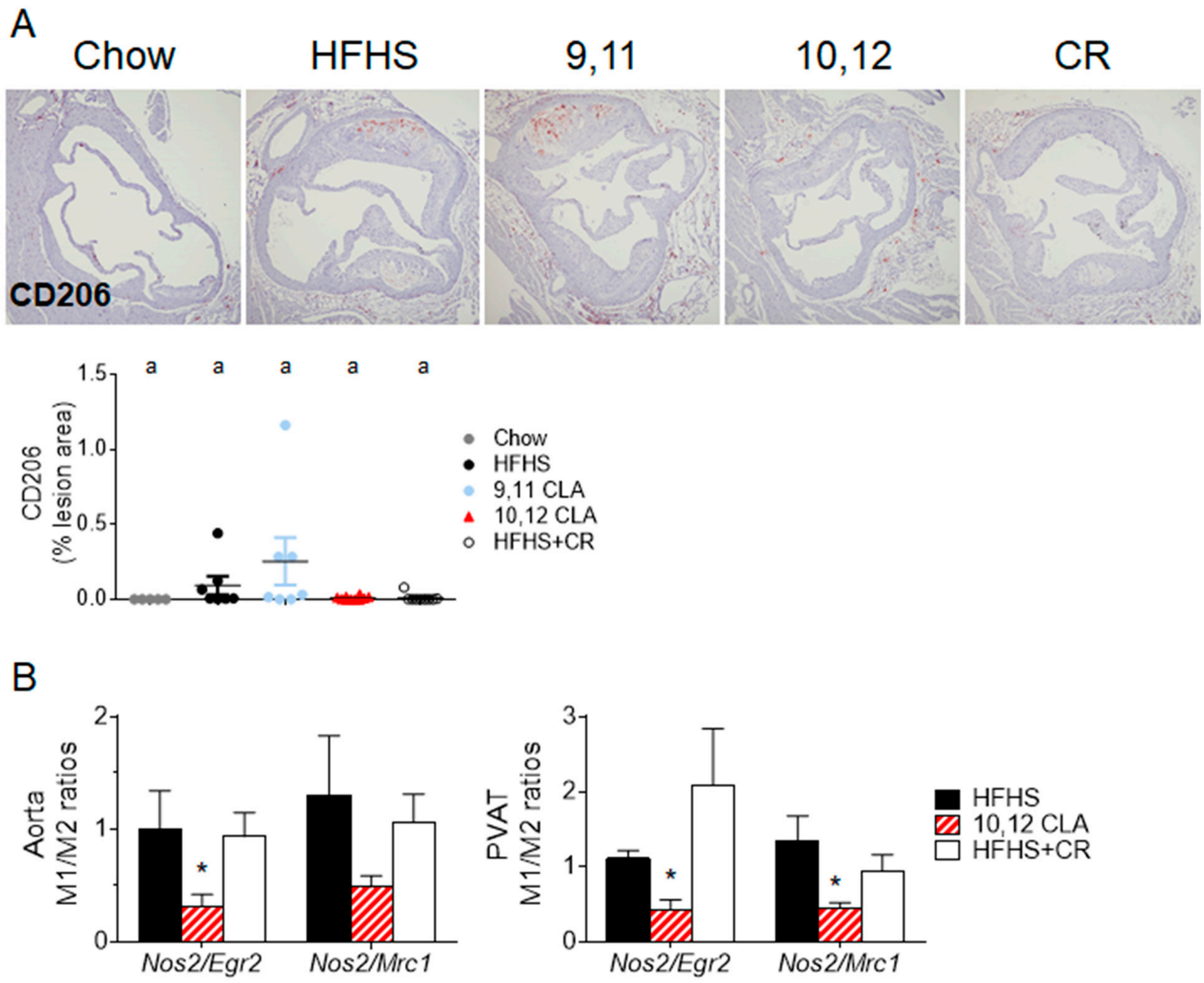

Figure S2. 10,12 CLA decreases the ratio of M1/M2 macrophages. *Ldlr*$^{-/-}$ mice were fed either chow or a HFHS diet for 12 weeks, then continued on the indicated diets for an additional 8 weeks. (A) Sections through the aortic sinus of the heart were stained with a polyclonal CD206 antibody, and staining was quantified. (B) RT-PCR was performed on cDNA from thoracic aortas and the surrounding PVAT. n = 8 mice/group. Data are presented as mean ± SEM. *P<0.05 from HFHS control.

Figure S3.

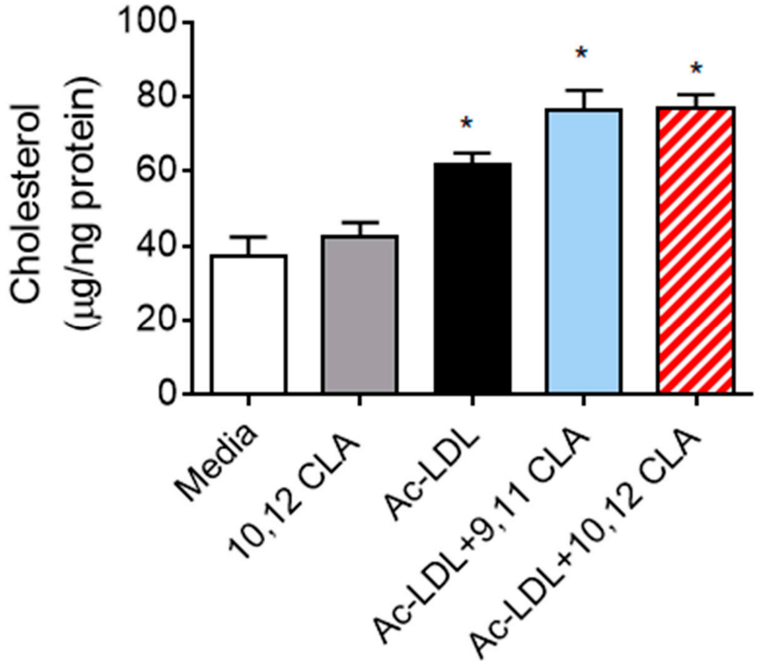

Figure S3. CLA isomers do not alter the uptake of cholesterol by bone marrow-derived macrophages. Non-polarized BMDMs were cultured with acetylated-LDL (50 µg/mL) for 24 hours, with or without co-treatment with 9,11 or 10,12 CLA (100 µM). CLA isomers were conjugated to fatty acid-free albumin at a 3:1 (fatty acid:albumin) molar ratio. n = 3. $p<0.05$ from media control.

References

[1] den Hartigh, LJ, Wang, S, Goodspeed, L, et al., Metabolically distinct weight loss by 10,12 CLA and caloric restriction highlight the importance of subcutaneous white adipose tissue for glucose homeostasis in mice, PLoS One, 2017;12:e0172912.
